# A One-Step Immunostaining Method to Visualize Rodent Muscle Fiber Type within a Single Specimen

**DOI:** 10.1371/journal.pone.0166080

**Published:** 2016-11-04

**Authors:** Shoko Sawano, Yusuke Komiya, Riho Ichitsubo, Yasuyuki Ohkawa, Mako Nakamura, Ryuichi Tatsumi, Yoshihide Ikeuchi, Wataru Mizunoya

**Affiliations:** 1 Department of Bioresource Sciences, Faculty of Agriculture, Kyushu University, Fukuoka, Japan; 2 Division of Transcriptomics, Medical Institute of Bioregulation, Kyushu University, Fukuoka, Japan; 3 CREST, JST, Saitama, Japan; 4 Department of Food Nutrition, Fukuoka Women's Junior College, Dazaifu, Japan; University of Minnesota Medical Center, UNITED STATES

## Abstract

In this study, we present a quadruple immunostaining method for rapid muscle fiber typing of mice and rats using antibodies specific to the adult myosin heavy chain (MyHC) isoforms MyHC1, 2A, 2X, and 2B, which are common marker proteins of distinct muscle fiber types. We developed rat monoclonal antibodies specific to each MyHC isoform and conjugated these four antibodies to fluorophores with distinct excitation and emission wavelengths. By mixing the four types of conjugated antibodies, MyHC1, 2A, 2X, and 2B could be distinguished within a single specimen allowing for facile delineation of skeletal muscle fiber types. Furthermore, we could observe hybrid fibers expressing MyHC2X and MyHC2B together in single longitudinal muscle sections from mice and rats, that was not attained in previous techniques. This staining method is expected to be applied to study muscle fiber type transition in response to environmental factors, and to ultimately develop techniques to regulate animal muscle fiber types.

## Introduction

Skeletal muscle tissue is composed of thousands of muscle fibers, and the contractile and metabolic properties of skeletal muscle tissues depend on their fiber type composition. There are mainly two fiber types: type 1 fibers (slow-twitch oxidative, red muscle) and type 2 fibers (fast-twitch glycolytic, white muscle). Type 1 fibers contain more mitochondria, possess a high oxidative capacity, and are resistant to fatigue. Meanwhile, type 2 muscle fibers show high rates of glycolytic metabolism and fatigue easily. As a result, muscles enriched in type 1 fibers, such as the soleus, typically perform sustained and tonic contractile activities, like postural tension, while muscles enriched in type 2 fibers, such as the extensor digitorum longus (EDL), are typically involved in intense and rapid activities of short duration. In human vastus lateralis muscles collected from a total of 418 Caucasians, the lowest and highest proportion of type 1 fibers were 15% and 85%, and the coefficients of variation (CV) reached approximately 30% [1], suggesting that there is a large variation in the composition of muscle fiber types between individuals. Overall, fiber type composition affects exercise performance, fatigue resistance, and metabolic capacity in humans [2]. Furthermore, animal model studies demonstrated a strong relationship between muscle fiber type and the development of diabetes and obesity [3][4]. Meanwhile, certain diseases can interfere with the composition or distribution of muscle fiber types, which can subsequently result in clinical manifestations [5]. Thus, elucidating the mechanism of muscle fiber type regulation would likely enhance our understanding of human metabolic disorders, exercise performance, and skeletal muscle diseases.

Myosin, a molecular motor with ATPase activity that generates contractile force through the consumption of ATP, is a predominant and key component of skeletal muscle proteins. The myosin molecule is comprised of a hexamer consisting of two identical myosin heavy chain (MyHC) subunits and four light-chain subunits. The catalytic domain of myosin, which is responsible for both ATP hydrolysis and interactions with actin, is located within the MyHC subunits [6]. To date, four predominant MyHC isoforms have been identified in adult rodent skeletal muscles: MyHC1, 2A, 2X, and 2B [7]. In general, each muscle fiber (muscle cell) expresses only one MyHC isoform. MyHC1 is expressed in type 1 muscle fibers. Meanwhile, type 2 fibers are subdivided into type 2A, 2X, and 2B muscle fibers, which preferentially express MyHC2A, 2X, and 2B, respectively. Notably, type 2A and 2X fibers exhibit intermediate contractile characteristics of type 1 and type 2B fibers. Although type 2X fibers are sometimes defined as fast-twitch glycolytic fibers, type 2B fibers have an even stronger fast-twitch glycolytic phenotype than these fibers [8][9][10].

Myosin ATPase staining [11] is a common and conventional procedure that has been widely adopted as the standard method for muscle fiber typing in skeletal muscle especially in clinical-pathological testing [12]. However, while this staining method, which is dependent upon the pH lability of each MyHC isoform, can be utilized to distinguish fiber types 1, 2A, and 2X, it is unable to distinguish between types 2X and 2B. Furthermore, because this procedure requires the preparation and comparison of multiple successive cryosections (typically, at least 3 sections are required for preincubation at pH 4.3, 4.6, and 10.4, respectively), it is very time consuming.

In previous studies, immunohistochemistry analyses using monoclonal antibodies specific to various isoforms of MyHC have been employed to distinguish fiber types with high levels of specificity [13][14]. Indeed, multicolor imaging is increasingly used in biological assays, particularly in immunostaining. Previously, mouse monoclonal anti-MyHC antibodies with isotype-specific secondary antibodies [15][16] were utilized to attain multiple staining of a single fiber cross-section. Still it is impossible to stain MyHC2X and MyHC2B simultaneously because they are of the same IgM subclass and in principle there is no secondary antibody that can distinguish the same IgM subclass antibodies. Furthermore, IgM antibodies are far less robust than IgGs. Many IgM mAbs are irreversibly denatured by freeze-drying [17]. IgM mAbs are also prone to aggregation after prolonged storage at 4°C [18]. In our experience, the reactivities of stored anti-MyHC2B and anti-MyHC2X were substantially lower than those of other monoclonal IgG antibodies, despite identical storage conditions. Direct labeling of anti-MyHC antibodies can overcome the overlap of a secondary antibody, and up to now MyHC1, 2A and 2B appears to be distinguished by fluorophore-conjugated primary antibodies [19]. Nonetheless, the labeling and handling of IgM must be still challenging because of their lability. Therefore, there is a benefit of producing new anti-MyHC antibodies which subclass are stable IgG.

**Fig 1:**
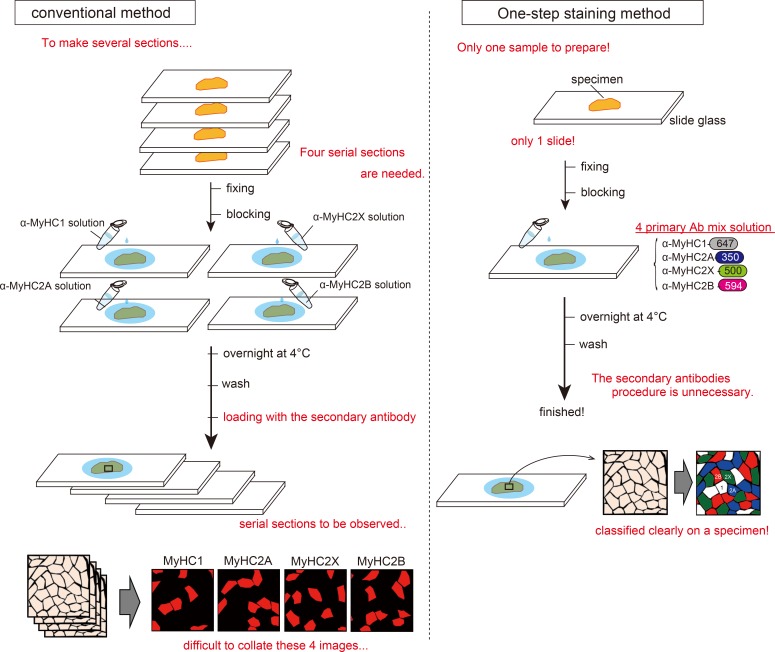
Scheme of our staining method and the conventional immunostaining method. The scheme in the panel on the left illustrates the conventional staining method. While this technique is widely used, it requires the production of several serial tissue sections to verify the distribution of each myosin heavy chain (MyHC) isoform. Hence, this method is time-consuming and laborious. Furthermore, multiple immunostaining of one section is difficult as all conventional anti-MyHC antibodies are mouse monoclonal antibodies. Meanwhile, the most time-consuming and laborious step in this process is to collate the serial sections, as it is challenging to locate the exact same regions within multiple large muscle cross-sections under a microscope. In contrast, our method is a very simple method. The staining is applied to individual tissue sections, and no secondary antibodies are required. It is therefore a highly efficient method for skeletal muscle fiber typing.

Thus, in this study, we generated new monoclonal IgG antibodies specific to each respective MyHC isoform, and utilized these antibodies for one-step multiple immunofluorescence imaging of rodent muscle tissues. Using this approach, which is depicted in Fig 1, we successfully generated fluorescent images of rodent muscle cross-sections labeled simultaneously with four fluorophore-conjugated antibodies with distinct absorption spectra, including a far-red wavelength and common RGB colors. Furthermore, we show that our method can be applied to the staining of cultured cells, for which the preparation of serial sections is not possible.

## Materials and Methods

### Animals

Animals were purchased from commercial suppliers, as described in each experiment below. Animals were housed in standard cages in an animal room at 22 ± 2°C and 50 ± 10% humidity with an artificial lighting system and 12-h light-dark schedule. Animals were fed a commercial diet (Type CRF-1 of Oriental Yeast Co., Ltd., Tokyo, Japan) *ad libitum* and allowed free access to water. All animal experiments were conducted in strict accordance with the recommendations in the Guidelines for Proper Conduct of Animal Experiments published by the Science Council of Japan, and with the ethical approval of the Animal Care and Use Committee of Kyushu University (approval no. A22-148, A24-138 and A26-081).

### Production of rat monoclonal anti-MyHC antibodies

To enable simultaneous staining of the four MyHC fiber types, we firstly produced rat monoclonal anti-MyHC antibodies specific to each isoform. For monoclonal antibody production, we utilized the rat lymph node method established by Sado *et al*. [20][21] in which enlarged medial iliac lymph nodes are used as a source of B cells for the generation of hybridomas, resulting in highly efficient and rapid antibody production.

MyHC1, 2A, 2X, and 2B are relatively large proteins in mice, encoding between 1935–1942 amino acids and having a molecular weight between 200–220 kDa. MyHC is comprised of a head (catalytic, motor) domain, which is generally at the N-terminus, followed by a neck domain, to which the light chains bind, and a C-terminal tail domain that dimerizes with an identical heavy chain via the formation of coiled-coil helical regions [22]. To obtain highly specific antibodies, we selected a 29-amino acid sequence within the tail domain that exhibits high levels of amino acid sequence identity among the four MyHC isoforms, with the exception of one amino acid, and prepared synthetic peptides mimicking these regions as shown in Table 1. Each peptide contained a distinct amino acid at the 19th position from the N-terminus. Specifically, MyHC1 contained a leucine (L), MyHC2A contained a methionine (M), MyHC2X contained an isoleucine (I), and MyHC2B contained a valine (V) at this position. Based on BLAST analysis, this amino acid sequence exhibits no homology to other proteins. Although it is generally preferable to select a sequence that has as many different amino acids as possible, we chose to utilize a sequence that was similar among the proteins to ensure that each antibody exhibited similar levels of accessibility to intracellular MyHC. We also predicted that protein conformational changes due to interactions with other proteins would be less likely in this tail domain region because coiled-coil helical regions were already formed. To allow for conjugation with carrier peptides, such as bovine serum albumin (BSA) and keyhole limpet hemocyanin (KLH), we designed each antigen to encode a cysteine (C) at the C-terminus. These peptide antigens were synthesized by Sigma-Aldrich (St. Louis, MO, USA).

**Table 1:** Synthesized peptides as an antigen for MyHC1, 2A, 2X or 2B.

	antigen amino acids sequence (29 a.a.)	amino acids region
MyHC1	C-ELESSQKEARSLSTELFKLKNAYEESLEH	1470–1498
MyHC2A	C-ELEASQKEARSLGTELFKMKNAYEESLDQ	1473–1501
MyHC2X	C-ELEASQKESRSLSTELFKIKNAYEESLDH	1473–1501
MyHC2B	C-ELEASQKESRSLSTELFKVKNAYEESLDQ	1466–1494

A cysteine (C) was linked with each antigen sequence to bind with a carrier peptide such as KLH and BSA.

To conjugate synthesized peptide antigens containing cysteine to carrier peptides, the following steps were needed. Briefly, 20 mg/mL carrier peptides in phosphate buffer were desalted with a PD-10 column, and then 15 mg/mL MBS (Sigma-Aldrich, St. Louis, MO, USA) was added to the desalted carrier peptides and stirred for 30 min. After column desalting of the mixed solution and doubling dilution, 5 mg/mL peptide antigen was added to the solution for the conjugation reaction. After adjusting to pH 7.0 with 0.1 M NaOH, the solution was stirred for 3 h. The conjugated peptide solution was stored at -20°C before use.

To generate rat monoclonal antibodies specific to each MyHC isoform, the hind footpads of 10-week-old female WKY/NCrlCrlj rats (Charles River Laboratories Japan, Kanagawa, Japan) were injected with 300 μL of an emulsion containing 120 μg of Freund’s complete adjuvant and the appropriate KLH-conjugated MyHC protein (3–10 mg/mL). After approximately 2 weeks, the cells from the lymph nodes of rats immunized with each respective antigen were fused with mouse myeloma SP2/0-Ag14 cells at a ratio of 5:1 in a 50% polyethylene glycol (PEG1500; Roche, Basel, Switzerland) solution. The resulting hybridoma cells were seeded onto 96-well plates and cultured in HAT selection medium [hybridoma SFM medium (Invitrogen, Waltham, MA, USA); 10% fetal bovine serum (FBS); 1 ng/mL interleukin 6 (IL-6; R&D Systems, Inc., Minneapolis, MN, USA); 100 μM hypoxanthin; 0.4 μM aminopterin; 16 μM thymidine]. Seven days post-fusion, the hybridoma supernatants were screened by enzyme-linked immunosorbent assay (ELISA) against the BSA-conjugated MyHC peptides. Briefly, 2 μg/mL of BSA-conjugated MyHC1, 2A, 2X, or 2B peptide suspended in 20 mM in phosphate buffer (pH 7.0) was adsorbed to the surface of 96-well flexible microplates (Thermo Fisher Scientific, Waltham, MA, USA) by incubating overnight at 4°C. To prevent non-specific binding, the plates were then blocked with 1% BSA in phosphate-buffered saline (PBS). Hybridoma supernatants were added to the wells, incubated for 1 h at room temperature, and washed three times with distilled water. The plates were then incubated for 30 minutes at room temperature with horseradish peroxidase-conjugated anti-rat IgG antibody (Sigma-Aldrich) diluted in 1% BSA-PBS 1:20,000. After washing three times with distilled water, immunoreactivity was visualized via peroxidase-based enzyme reactions with 3,3',5,5'-tetramethylbenzidine (TMBZ; Wako Pure Chemical Industries Ltd., Osaka, Japan). Hybridomas were subsequently monocloned by limiting dilution culture. Positive clones were subcloned and rescreened by ELISA until we obtained single positive clones. We also tested the immunoreactivity of certain positive clones by immunostaining of rodent muscle cross-sections.

Ultimately, we produced hybridomas against each MyHC isoform, and the specific immunoglobulin class of each hybridoma was determined using an RMT1 rat isotyping kit (AbD Serotec, Oxford, UK). These analyses indicated that the monoclonal antibodies (MAb) specific to MyHC1 (clone 4B51E8) and MyHC2A (clone 8F72C8) are rat IgG2a (κ) antibodies, while the MyHC2X-specific (clone 6F12H3) and MyHC2B-specific (clone 2G72F10) MAbs are IgG1 class antibodies (κ). To obtain concentrated MyHC antibody solutions, the positive clones were cultured until the hybridoma supernatants contained abundant antibodies. The respective supernatants were then purified using HiTrap Protein G HP columns (GE Healthcare, Little Chalfont, UK). The produced hybridomas were split and stored using backup service by Interuniversity Bio-Backup Project for Basic Biology (IBBP) under National Institute for Basic Biology (Okazaki, Japan) for future distribution.

### Fluorophore conjugation of MyHC antibodies

The purified anti-MyHC1, anti-MyHC2A, anti-MyHC2X, and anti-MyHC2B antibodies were conjugated to the Alexa Fluor 647, Alexa Fluor 350, Fluorescein, and AnaTag™ HiLyte™ Fluor 594 fluorophores, respectively. Conjugation reactions were carried out according to the manufacturer’s instructions for the Alexa Fluor 647 Monoclonal Antibody Labeling Kit (Life Technologies, Carlsbad, CA, USA), the Alexa Fluor 350 Antibody Labeling Kit (Life Technologies), the Fluorescein Labeling Kit-NH_2_ (Dojindo Laboratories, Kumamoto, Japan), and the AnaTag™ HiLyte™ Fluor 594 Microscale Protein Labeling Kit (AnaSpec, Inc., Fremont, CA, USA).

### Muscle sections

Male F344 rats (5 weeks old) and male C57BL/6J mice (11 weeks old) were purchased from Charles River Laboratories Japan. The muscle complexes of the gastrocnemius, plantaris, and soleus muscles were rapidly dissected from rats or mice after euthanasia by cervical dislocation under sevoflurane inhalation anesthesia. In addition, we used rat muscles obtained in previous studies for these experiments; the plantaris [23] and soleus [24] muscles obtained from 12-week-old male Wistar rats and stored at -80°C without any treatment, which were thawed and handled as fresh muscle samples. Meanwhile, longitudinal cryosections were also prepared from the plantaris muscles. For cryosectioning, muscle tissues were immediately placed in a cryodish (#CRYO DISH No.2; Shoei Works Co., Ltd., Tokyo, Japan) containing OCT-compound, and snap-frozen in isopentane cooled with liquid nitrogen. Ten micrometer sections from each block were collected onto Matsunami adhesive silane (MAS)-coated glass slides (S9226; Matsunami Glass Ind., Ltd., Osaka, Japan) and stored at -30°C until use.

### Conventional immunostaining method

Slides containing tissue sections were immersed in hot PBS solution in a tall Coplin polypropylene staining jar, and steamed in a food steamer (SP-4138W; Twinbird Corporation, Tsubame City, Japan) for 5 minutes. The PBS solution containing the sections was allowed to cool for at least 30 min at room temperature, and the slides were then incubated in 1.0% Triton X-100 in PBS at room temperature for 10 minutes. Subsequently, the sections were incubated at room temperature for 1 h in filtered blocking buffer (2% donkey serum, 1% BSA, 0.1% cold fish skin gelatin, 0.1% Triton X-100, 0.05% Tween 20, 0.05% sodium azide, 100 mM glycine, 0.1 mg/mL unconjugated AffiniPure Fab Fragment Donkey anti-rat IgG (Jackson ImmunoResearch Laboratories Inc., West Grove, PA, USA), and 0.01% avidin in 10 mM PBS). Hybridoma supernatants or the purified MAb antibodies 4B51E8, 8F72C8, 6F12H3, and 2G72F10 (diluted to 2 μg/mL in filtered primary antibody diluent buffer) were used as primary antibodies against MyHC1, 2A, 2X, and 2B, respectively. Furthermore, we used the following commercially available antibodies as conventional anti-MyHC antibodies: MyHC1 MAb (2 μg/mL) (M8421; Sigma-Aldrich), hybridoma supernatant from MyHC2A MAb [2F7; Developmental Studies Hybridoma Bank (DSHB) at the University of Iowa, Iowa City, IA, USA], hybridoma supernatant from the MyHC2X MAb clone (6H1; DSHB), and hybridoma supernatant from MyHC2B MAb clone BF-F3 (generous gift from Dr. Naoki Ito, National Center of Neurology and Psychiatry, Kodaira, Japan). The primary antibody diluent buffer contained 1% BSA, 0.1% cold fish skin gelatin, 0.5% Triton X-100, and 0.05% sodium azide in 10 mM PBS. Each antibody was loaded onto a specimen and incubated overnight at 4°C. The MyHCs were then treated with Biotin-SP-conjugated AffiniPure Fab Fragment Donkey anti-rat IgG (1:200 dilution; Jackson ImmunoResearch Laboratories Inc., West Grove, PA, USA) in filtered secondary antibody dilution buffer (0.05% Tween 20 in 10 mM PBS) for 1 h at room temperature. After washing with Tris-buffered saline containing 0.1% Tween 20 (TBS-T), sections were incubated with a 1:500 dilution of CF594 conjugated-streptavidin (Biotium, Hayward, CA, USA) in filtered secondary antibody dilution buffer for 20 min at room temperature. Coverslips were then mounted on the samples with Fluorescent Mounting Medium (S3023; Dako, Glostrup, Denmark).

### One-step staining method (our staining method)

Glass slides containing tissue sections were placed in a tall Coplin polypropylene staining jar filled with hot PBS solution and steamed in a food steamer for 5 minutes. After heating, the specimens were allowed to cool for 30 min at room temperature, and the slides were placed in a separate tall Coplin staining jar containing 1.0% Triton X-100 in PBS and incubated at room temperature for 30 min. The slides were then blocked by incubating in filtered blocking buffer (1% BSA, 0.1% cold fish skin gelatin, 0.1% Triton X-100, 0.05% Tween 20, 0.05% sodium azide, and 100 mM glycine in 10 mM PBS) for 1 h at room temperature. Meanwhile, the antibody solution was prepared by mixing the four conjugated antibody solutions containing the antibodies specific to the individual MyHC isoforms (diluted 1:100 or 1:200) in filtered primary antibody diluent buffer. After the blocking treatment, the antibody solution was loaded onto each specimen and incubated overnight at 4°C. The following day, the slides were washed with TBS-T, and coverslips were mounted on the specimens with Fluorescent Mounting Medium (S3023; Dako).

### Muscle fiber isolation

We isolated and plated single muscle fibers from the muscles of rats or mice, according to an established protocol [25]. Male Wistar rats (4 weeks old) or male C57BL/6J mice (4 weeks old) were purchased from Charles River Laboratories Japan (Kanagawa, Japan). The soleus, plantaris, or extensor digitorum longus (EDL) muscles were harvested from rats and mice and suspended in physiological rodent saline (PRS: 138 mM NaCl, 2.7 mM KCl, 1.8 mM CaCl_2_, 1.06 mM MgCl_2_, 12.4 mM HEPES, and 5.6 mM glucose, pH 7.3). All connective tissues, fat, blood vessels, and other non-muscle tissues were then removed using micro scissors under a dissecting microscope. Next, muscles were incubated in collagenase solution [PRS containing 0.2% collagenase Type 1 (Worthington Biochemical Corp., Lakewood, NJ, USA), 0.1% elastase (Worthington), 0.0625% protease from *Streptomyces griseus* (Sigma-Aldrich), 0.033% dispase (Invitrogen), and 10% FBS (Invitrogen)] for 90 min at 37°C with 5% CO_2_ to digest connective tissues. After collagenase digestion, muscles were transferred to proliferation medium [PM: high glucose Dulbecco’s Modified Eagle Medium (DMEM, Life Technologies) containing 10% FBS, 1% antibiotic-antimycotic mix (Life Technologies), and 0.1% gentamycin (Life Technologies)] using a flame-polished wide-bore Pasteur pipette. To dissociate the muscles into single muscle fibers, tissues were triturated with pipettes of decreasing diameter. Fibers were allowed to settle and were then washed. This gravity sedimentation and washing was repeated two additional times. Isolated mouse and rat muscle fibers were transferred onto MAS-coated glass slides with PM, steamed for 5 min, and subjected to immunostaining on a slide glass from the subsequent step after steam treatment. All instruments utilized in this protocol were sterilized prior to use.

### Image acquisition analysis

All immunostained samples were visualized using a Leica DMI6000B-AFC fluorescence microscope system (Leica Microsystems, Wetzlar, Germany) equipped with a charge-coupled device (CCD) camera (DFC365FX; Leica). Samples were excited with 100 W Hg lamp, and the excitation and emission signals were filtered using the following filter cubes: Y5 (for Alexa Fluor 647), A4 (for Alexa Fluor 350), L5 (for Fluorescein), and TX2 (for AnaTag™HiLyte™Fluor 594). All filter cubes were manufactured by Leica Microsystems. Images were observed with a 10×/0.30 PH1 HCX PL FLUOTAR objective lens (506507; Leica), and the Tile Scan feature was utilized to obtain whole images of each cross section. This function also enabled us to scan multiple partial images from a specimen and stitch them together to form a complete large image (excerpt from “Confocal Application Notes VOl.5 July 2010). Because the camera used was a highly sensitive monochrome camera, colors were painted on the images as pseudocolors using a microscope image analysis program (LAS AF, version 3.1.0; Leica). Pseudocolors that were closest to the emission wavelength were chosen, with the exception of Alexa Fluor 647 fluorophore, which emits in the near-infrared spectrum and was shown as white. Image analysis and optimization were performed using Adobe PhotoShop CS6 (version 13.0 x32).

### Western blotting

The EDL and soleus muscles from 8-week-old Wistar rats (Kyudo, Tosu, Japan) were ground to a powder using a mortar and pestle cooled with liquid nitrogen. The powdered muscles (approx. 50 mg) were homogenized with a motor-driven small pestle in 0.1 M Tris-HCl buffer (pH 8.0) containing 10% SDS, 40 mM DTT, 5 mM EDTA, and a 1:100 dilution of Protease Inhibitor Cocktail for Use with Mammalian Cell and Tissue Extracts (Nacalai Tesque, Inc., Kyoto, Japan), and then heated in boiling water for 3 min. The total protein concentration of each sample was assayed using the Pierce BCA Protein Assay Reagent (Thermo Scientific, Waltham, MA, USA), with BSA as a standard. The samples were then diluted in 2× sample buffer (100 mM DTT, 4.0% SDS, 0.16 M Tris-HCl (pH 6.8), 43% glycerol, and 0.2% bromophenol blue) and dH_2_O to final protein concentrations of 50 ng/μL in 1× sample buffer. The protein samples were stored at -80°C until use for assays. Mixtures of EDL and soleus protein samples were subjected to high-resolution SDS-polyacrylamide gel electrophoresis (PAGE) for the separation of MyHC isoforms, as described in detail by Mizunoya *et al*. [26]. Briefly, the 8% acrylamide (the acrylamide/Bis ratio was 99:1) gel containing 35% (v/v) glycerol was used. After protein samples (250 ng protein/sample) were loaded, electrophoresis was performed at a constant voltage of 140 V for 22 h at 4°C. MyHC isoforms migrate at different rates (MyHC1 > 2B > 2X > 2A) allowing for efficient differentiation of these proteins. The electrophoretically-separated MyHC isoforms were then transferred to Amersham Hybond LFP 0.2 polyvinyl fluoride (PVDF) membranes (GE Healthcare) by exposing to 38 V (constant voltage) for 4 h at room temperature. The membranes were stained with the colloidal gold stain from the PROTOGOLD Kit (BBI solutions, Cardiff, UK), blocked with 5% skim milk diluted in TBS-T for 45 min at room temperature, and blotted by incubating overnight with the primary antibody mix [2 μg/mL anti-MyHC1 or anti-MyHC2X, or 1 μg/mL anti-MyHC2A or anti-MyHC2B in Can Get Signal solution 1 (Toyobo Co., Life Sciences Dept., Osaka, Japan)] at 4°C with gentle agitation. After washing three times in TBS-T for 10 min at room temperature, the membranes were incubated for 1 h at room temperature with a 1:5000 dilution of horseradish peroxidase-conjugated donkey anti-rat IgG (Jackson ImmunoResearch Inc.) in Can Get Signal solution 2 (Toyobo), and again washed three times with TBS-T for 10 min at room temperature. The blots were developed by enhanced chemiluminescence (ECL Western Blotting Reagents, GE Healthcare), and images were captured using a Fusion SL-4 chemiluminescence imager (Vilber Lourmat, Marne La Vallée, France).

## Results

### Verification of the specificity of the MyHC antibodies

**Fig 2:**
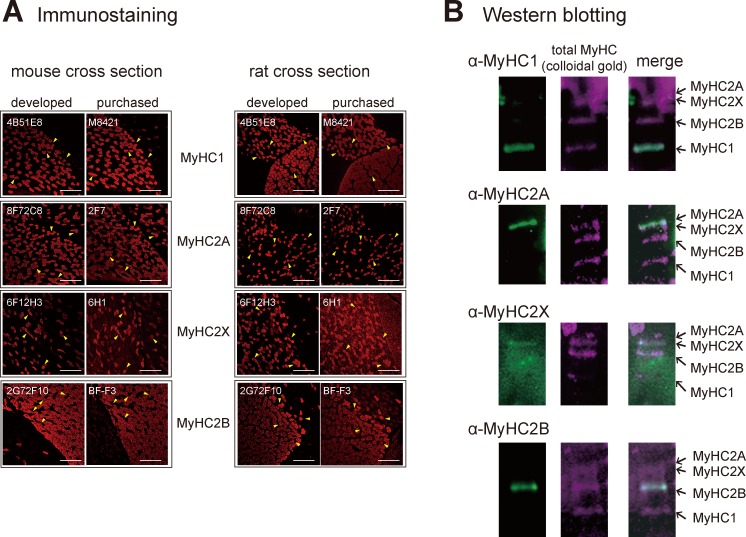
Evaluation of the validity of the myosin heavy chain (MyHC) monoclonal antibodies. (A) We compared the efficacy of our newly developed antibodies with commercially available antibodies for immunofluorescence staining analyses of serial sections of skeletal muscle tissues. The images on the left show the staining patterns of each MyHC isoform in mouse sections, while the images on the right show the staining patterns in rat cross-sections. The distributions of MyHC1, MyHC2A, MyHC2X, and MyHC2B, which were stained with our newly developed antibodies, were nearly identical to those observed after staining with commercially available antibodies. Yellow arrowheads indicate representative examples of MyHC-positive fibers. The bars indicate 250 μm. The characters described at the upper-left of each image denote clone names. (B) Western blotting analysis using our newly developed monoclonal anti-MyHC antibodies. Mixtures of rat soleus and extensor digitorum longus muscle homogenates were separated by 8% SDS polyacrylamide gel electrophoresis [26], and blotted on a polyvinyl fluoride (PVDF) membrane. The specific immunoreactivities were confirmed by merging colloidal gold-stained total MyHC (pseudo colored magenta) and the bands detected by each antibody (pseudo colored green). Overlapping bands appear white in the merged images.

The newly-developed antibodies were utilized for indirect immunofluorescence labeling of cryostat sections of mouse and rat skeletal muscles. Our anti-MyHC antibodies reacted to the same muscle fibers as the conventionally used, commercially available anti-MyHC antibodies in both mouse and rat muscles (Fig 2A). We used rat serum that is thought to contain preimmune rat IgG as an isotype control. Any non-specific reaction by preimmune rat IgG was not detected, indicating specific reaction of our anti-MyHC antibodies (S1 Fig). These results indicate that our antibodies specifically recognize each MyHC isoform. Moreover, our antibodies can also be effectively used for Western blot analyses. A clear, single band was observed at the position of each respective MyHC isoform after separation by modified SDS-polyacrylamide gel electrophoresis (PAGE) [26], indicating specific reactions to the target MyHC isoforms (Fig 2B).

### Simultaneous four-color visualization of MyHC isoforms

**Fig 3:**
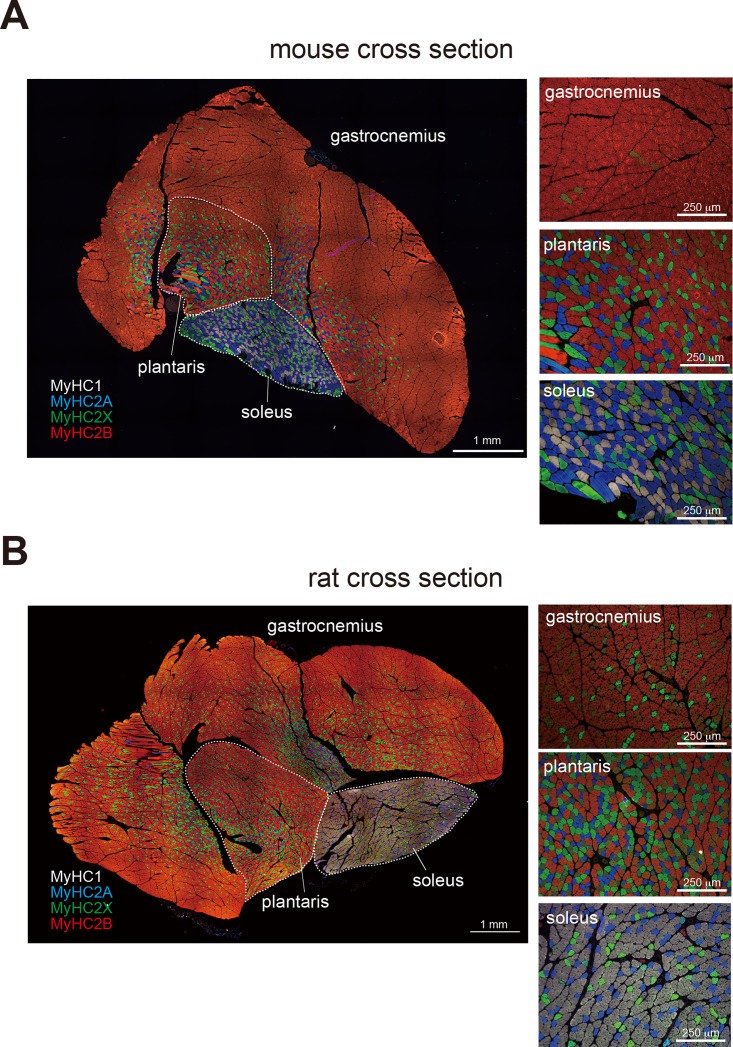
Mouse and rat cross-sections stained using the staining method. For these experiments, a single skeletal muscle section from a mouse (A) or a rat (B) was used. Cross-sections were obtained from calf muscles, including the gastrocnemius, plantaris, and soleus muscles, and immunopositive MyHC isoforms were visually classified as MyHC1 (white), MyHC2A (blue), MyHC2X (green), and MyHC2B (red). Images were taken with a 10× objective lens and assembled seamlessly using a tiling program, and high-resolution images covering whole muscle cross-sections were obtained. The panels on the right contain images that were magnified to determine the differences in distribution of the MyHC isoforms by examining the prevalence of each color. Bars indicate 1 mm in the whole muscle image, and 250 μm in the magnified images.

In general, it is difficult to perform immunostaining using multiple primary antibodies derived from the same species because a secondary antibody can not distinguish them. To overcome this problem, we conjugated our newly-developed MyHC antibodies to the following fluorophores: Alexa Fluor 647 was conjugated to the anti-MyHC1 antibody, Alexa Fluor 350 to anti-MyHC2A, Fluorescein to anti-MyHC2X, and HiLyte Fluor 594 to anti-MyHC2B. The emission peaks of these four fluorophores can be sufficiently discriminated using proper optical filters. As a result, our method is advantageous in that the usage of fluorophore-conjugated antibodies eliminates the necessity of secondary antibodies, and mixtures of the four antibodies can be utilized to stain and visualize a single sample. Indeed, this method enabled the simultaneous and clear visualization of the four MyHC isoforms in both mouse and rat skeletal muscle cross-sections (Fig 3). Images of stained mouse calf muscles (gastrocnemius, plantaris muscle, and soleus muscle) are included in Fig 3A, while images of rat muscles are presented in Fig 3B. All images were taken with a 10× objective lens and assembled seamlessly using the Tile Scan tiling program, resulting in the production of obtain high-resolution images covering whole muscle cross-sections. Because the camera was a highly sensitive monochrome camera, the colors were painted on the images as pseudocolors using a microscope image analysis program. Specifically, the colors closest to the emission wavelengths of the respective fluorophores were chosen for these pseudocolors, except for the Alexa Fluor 647-labeled antibody, which emits in the near-infrared spectrum and was shown as white.

The soleus muscles of both mice and rats, which compose the inner part of calf, predominantly expressed MyHC1 or MyHC2A, indicating that these muscles were comprised of slow-twitch or intermediate fibers. While the soleus muscles of the mice exhibited high levels of MyHC2A fibers, those of the rats were comprised predominantly of MyHC1 fibers, which is consistent with the results of a previous report [27]. Conversely, the plantaris muscles (adjacent to the soleus muscles) of both animals were composed primarily of MyHC2B fibers; however, there were also considerable numbers of MyHC2A and MyHC2X fibers, which exhibit intermediate characteristics, present in these tissues. Lastly, while the gastrocnemius muscles from both rats and mice were composed primarily of MyHC2B fibers, the distribution of this protein was not uniform. The fast-twitch MyHC2B fibers were more abundant in superficial regions than in deeper tissues, which is consistent with observations made in a previous study [28]. The MyHC composition of the plantaris and gastrocnemius muscles was relatively similar between mice and rats. However, the gastrocnemius muscles from rats contained more MyHC2X fibers than those from mice, suggesting that the calf muscles of rats exhibit more slow-type characteristics than those of mice.

In this study, we examined seven different conditions to optimize the fixation procedure for the staining (S1 Table). We found that two heat treatments (steam and microwave) provided effective staining, whereas other fixation methods, such as paraformaldehyde or acetone treatment, totally abolished the antigenicity of the MyHC isoforms.

### This staining method can be utilized to detect alterations in muscle fiber types

**Fig 4:**
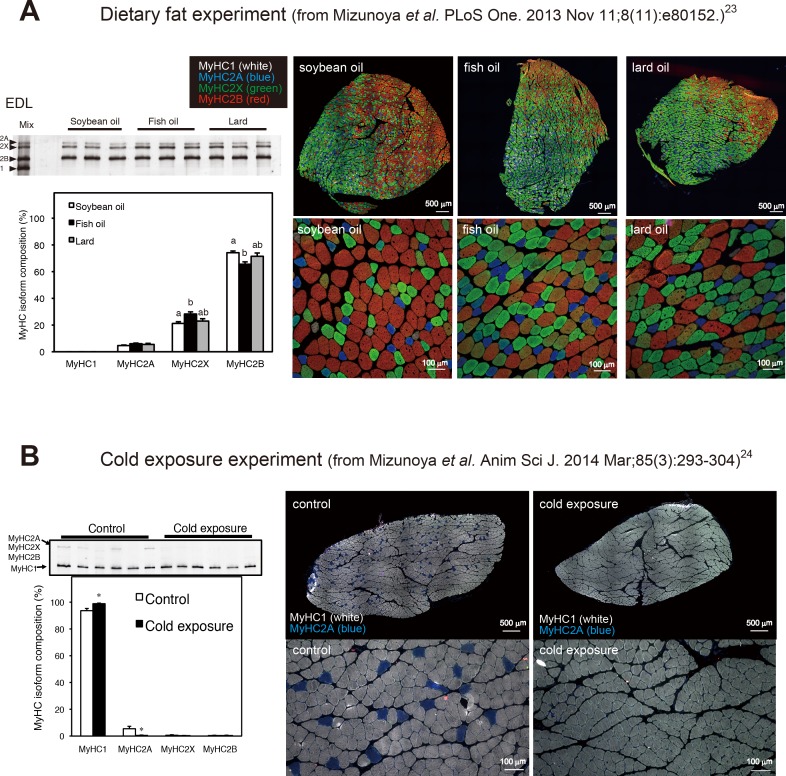
Practical applications of the staining method. One-step staining can provide additional data supporting the results of the electrophoretic detection of MyHC isoforms. (A) The dietary fat experiment: The electrophoretic image and the graph, published by Mizunoya *et al*. [23], contained in the panels at the left demonstrate that fish oil intake for four weeks induced significantly lower expression levels of the fast-type MyHC2B and higher expression levels of the intermediate-type MyHC2X protein in the extensor digitorum longus (EDL) muscles of rats than did the intake of soybean oil. Our one-step staining method succeeded in verifying the decrease in MyHC2B fibers and the increase in MyHC2X fibers in the plantaris muscles of fish oil-fed rats compared with soybean oil-fed rats. (B) The cold exposure experiment: The electrophoretic image and the graph, published by Mizunoya *et al*. [24], in the panels at the left show that cold exposure increased the prevalence of slow-type MyHC1 fibers in the soleus muscles of rats. Consistent with these findings, our staining method clearly depicts the predominance of MyHC1 in cross-sections of soleus muscles from these same cold-exposed rats. Bars indicate 500 μm in the whole images (upper) and 100 μm in the magnified images (bottom), respectively. Different superscripts indicate a significant difference between two groups (p < 0.05, one-way ANOVA; post hoc: Tukey–Kramer multiple-comparison test). *P < 0.05, compared to control group by unpaired Student’s *t*-test.

To examine whether our staining method could be applied to the evaluation of muscle fiber type transitions, we analyzed a panel of muscle samples obtained during two of our previous studies [23][24]. In these studies, we assessed alterations in muscle fiber types by SDS-PAGE analysis of muscle tissue homogenates. While this is an efficient method for obtaining a global picture of the expression levels of MyHC isoforms, the usage of homogenates does not allow for analyses of the distribution of MyHC isoforms within the muscle tissue or of muscle fiber morphology (fiber diameter or size). To address these questions, we utilized our staining method to examine samples obtained during these studies. In the first study, we had examined the effects of consumption of different dietary oils on the muscle fiber types present in rats and found that the EDL muscles (fast twitch fiber dominant) of animals that were fed fish oil exhibited significantly lower levels of MyHC2B and higher levels of MyHC2X expression than those of rats that were fed soybean oil [23]. Meanwhile, rats that were fed lard exhibited an intermediate MyHC composition between soybean oil-fed and fish oil-fed rats (Fig 4A left, SDS-PAGE results from published data [23]). Because there were no EDL samples remaining from this previous study, we chose to utilize the rat plantaris muscles, which exhibit a MyHC composition similar to that of EDL muscles, for our immunofluorescence analyses. As depicted in Fig 4A (right), there was a marked increase in the proportions of MyHC2X-positive fibers, and a concurrent decrease in the proportions of MyHC2B-positive fibers, in the plantaris muscles of fish oil-fed rats compared with those of soybean oil-fed rats. Here, all fibers of representative animal plantaris muscles (about 3000 fibers per muscle), which were close to average value of each group, were counted and shown in Table 2. These finding were therefore consistent with those previously obtained by SDS-PAGE analyses.

**Table 2:** Percent distribution of pure MyHC1, 2A, 2X, 2B-positive and hybrid fibers in the rat plantaris muscles, based on immunofluorescent analyses shown in Fig 4A (right).

	pure fibers				hybrid fibers			
	MyHC1	MyHC2A	MyHC2X	MyHC2B	1+2A	1+2X	2A+2X	2X+2B
soybean oil	6.4	14.7	33.6	25.2	0.0	0.0	5.6	14.5
fish oil	9.8	17.4	41.6	12.0	0.4	0.0	5.4	13.4
lard oil	8.8	18.4	51.1	12.3	0.0	0.0	1.8	7.5

In the subsequent study, we examined muscle samples from cold-exposed rats. Our SDS-PAGE analyses revealed that the soleus muscles (slow twitch fiber dominant muscle) from cold-exposed rats were composed primarily of MyHC1, while those from the control population expressed high levels of MyHC1 but also expressed MyHC2A (Fig 4B left, SDS-PAGE results from published data [24]). Likewise, the staining of cold-exposed and control soleus muscles clearly demonstrated that the cold-exposed tissues were composed entirely of MyHC1 fibers (Fig 4B right). All fibers of representative animal soleus muscles (about 3000 fibers per muscle), which were close to average value of each group, were counted and shown in Table 3.

**Table 3:** Percent distribution of pure MyHC1, 2A, 2X, 2B-positive and hybrid fibers in the rat soleus muscles, based on based on immunofluorescent analyses shown in Fig 4B (right).

	pure fibers				hybrid fibers			
	MyHC1	MyHC2A	MyHC2X	MyHC2B	1+2A	1+2X	2A+2X	2X+2B
control	94.9	4.0	0.0	0.0	1.0	0.0	0.0	0.0
cold exposure	99.9	0.0	0.0	0.0	0.1	0.0	0.0	0.0

### This staining method can be used for cell cultures

**Fig 5:**
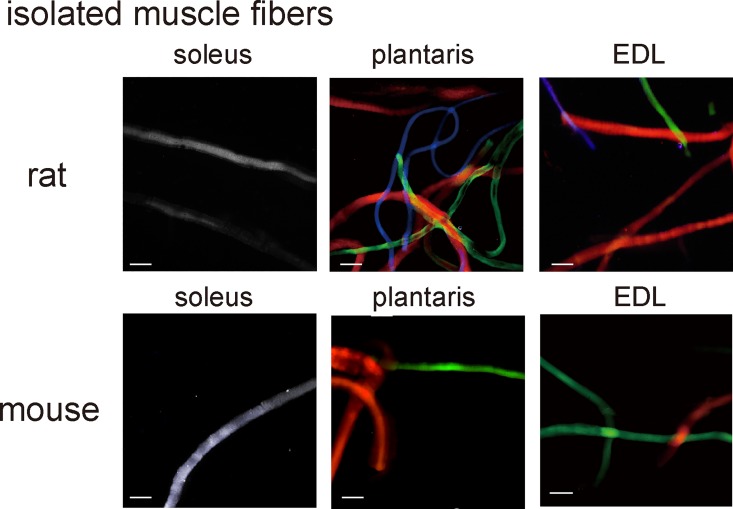
Practical applications of the staining method for cell cultures (isolated muscle fibers). Muscle fibers isolated from the soleus, plantaris, or extensor digitorum longus (EDL) muscles of mice or rats were stained using the staining method. All bars indicate 100 μm.

The cell culture is the important tool for life science research field. We found the present staining method can be used for immunostaining of cultured cells. We prepared the isolated muscle fibers from the skeletal muscle tissues of rats or mice, in which adult MyHC isoforms are present. The isolated fibers were allowed to adhere to an MAS-coated slide and subjected to our staining method in the same manner as the cryosections. Using this method, we could obtain the images that visualized MyHC isoforms in isolated fibers (Fig 5).

### Detection of hybrid fibers within longitudinal sections

**Fig 6:**
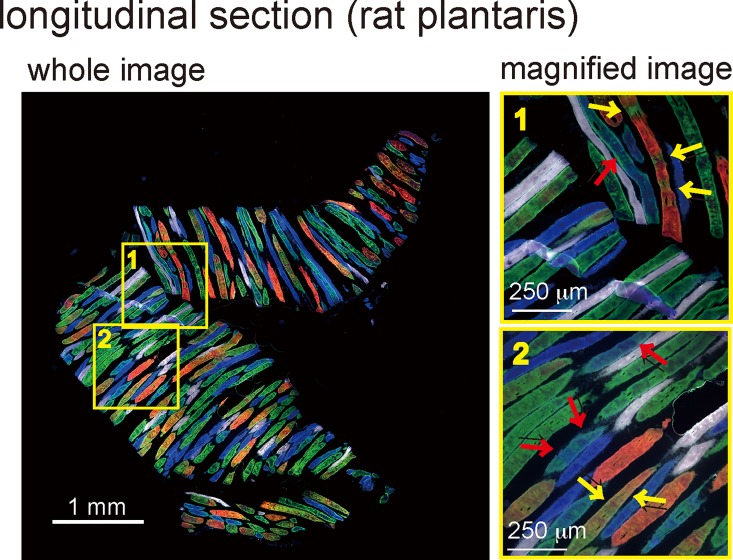
Practical applications of the staining method for analysis of longitudinal sections. A longitudinal section from a rat plantaris muscle was stained using the staining method. The whole image provides a global view of the MyHC isoforms present in the section. The magnified images in panels 1 and 2 show the presence of hybrid fibers comprised of MyHC2X (green) and MyHC2B (red), or of MyHC2A (blue) and MyHC2X (green), as indicated by yellow and red arrows, respectively. Bars indicate 1 mm in the whole image and 250 μm in the magnified images, respectively.

Longitudinal sections of rat plantaris muscles stained with the four MyHC isoforms detected several MyHC hybrid fibers as well as the distribution of these isoforms within individual muscle fibers. In the rat plantaris muscle depicted in Fig 6, several hybrid fibers consisted of MyHC2 (A, X, and B) isoforms. However, there were some variations in the hybrid patterns, e.g. striped pattern, overlapping pattern, and spotted pattern.

## Discussion

The activation/contraction pattern of skeletal muscle, e.g., exercise training [29], external electrical stimulation [30], mechanical unloading [31], paralysis due to spinal cord injury [32], denervation [33], cross-reinnervation (fast muscle reinnervated by a slow nerve and vice versa) [34], is the most well-known environmental factor that affects fiber type composition. In general, increased persistent muscle contraction leads to transition from fast-to-slow type fibers while decreased contraction leads to a slow-to-fast fiber type transition. Furthermore, hormonal status (particularly that of thyroid hormones [35] and of food components [23][36]) can also alter muscle fiber types. While these findings demonstrate that muscle fiber types can be readily altered, an efficient method for detecting this transformation did not exist. Our immunofluorescence-based fiber-typing method developed in this study enables rapid and detailed analyses of these phenomena, and will likely accelerate our understanding of the environmental factors that modify skeletal muscle fiber types.

Previously, Jackson *et al*. reported that bright green autofluorescence exist in a specific subset of skeletal muscle fibers that strongly resembles emission from green fluorescent protein (GFP) deriving from oxidative muscle fibers [37]. In our experiments, the anti-MyHC2X antibody was conjugated to the Fluorescein, which has an emission maximum of 512 nm, similar to GFP emission maximum of 509 nm. In our experiments, we prepared the negative control without primary antibodies, checked the level of autofluorescence, and confirmed the green fluorescence derived from conjugated antibody was much stronger than autofluorescence. Moreover, according to Jackson *et al*. [37], MyHC2A-positive fibers should have high level of green autofluorescence. Nevertheless, the immunostained green fluorescence positive fibers, which derived from anti-MyHC2X antibody, were different from MyHC2A-positive fibers in our results, suggesting green autofluorescence can be distinguished from the Fluorescein in our staining method.

In addition to immunofluorescence staining of MyHC isoforms in muscle sections, there are several methods for determining muscle fiber types. As mentioned in the RESULTS section, a simple and quick method for fiber typing involves the separation of MyHC isoforms by modified SDS-PAGE using a mini-gel system, as depicted in Fig 4 (left panels). In addition, a previous study found that the results of quantitative PCR (qPCR) analyses of muscle f**ib**er composition correlated with those obtained by immunohistochemistry, indicating that qPCR can also be utilized for muscle fiber typing [38]. Nonetheless, it is not possible to visualize the localization or distribution of the distinct MyHC isoforms within muscle tissues and muscle cells using these methods.

Great care must be taken regarding sample fixation when applying this staining method. There are many distinct approaches for the fixation of antigens, which can sometimes be combined with antigen retrieval treatment. Currently, a gold standard fixation method that is applicable to all antigens does not exist. For example, certain antibodies can bind to target antigens with limited fixation. Meanwhile, some fixation methods can result in high background staining. In previous reports, heat treatment eliminated the high background levels observed during staining of unfixed cryostat sections of mouse skeletal muscle and enabled easy detection of several proteins, including the scarce dystrophin protein, with murine monoclonal antibodies [39]. In this study, we examined several fixation methods and found that two heat treatments (steam and microwave) provided effective staining. Thus, heat pretreatment of samples is essential for our staining method. Furthermore, we found that an ordinary rice/vegetable steamer provided a highly reproducible and more convenient method for heating samples than boiling and microwaving, which was previously utilized by Tang *et al*. [40].

When examining only a few continuous serial cross-sections, the fiber distributions within these sections are typically quite similar. As a result, although the process is time-consuming and laborious, it is possible to match and collate images of the fibers in these serial sections. Conversely, there are typically, in principle, large differences in the fiber patterns among serial longitudinal sections of skeletal muscles. Our staining method enabled visualization of MyHC isoform distributions in a single longitudinal section. While hybrid fibers expressing two or more MyHC isoforms were suggested to exist in the skeletal muscles of small mammals [41][42][43], it was previously not possible to simultaneously visualize all the adult MyHC isoforms present in these fibers (especially MyHC2X and 2B). As a result, previous studies failed to fully characterize these fibers or their spatial distribution. Notably, our staining method developed in this study makes such staining and imaging possible. Indeed, our data indicate that MyHC isoforms localize in several patterns within rat and mouse muscles under sedentary and normal conditions.

In summary, we have developed a novel staining method for simultaneous detection of the four MyHC isoforms within primary rodent muscle tissues as well as in isolated single muscle fibers. We expect that this staining method will facilitate the characterization of the mechanisms of fiber type transition in response to environmental factors and, ultimately, the development of techniques to control animal muscle fiber types artificially. Due to similarities between our images and stained glass art works, we refer to this staining method as stained glass staining.

## Supporting Information

S1 Fig Isotype control. Slides containing muscle sections were immunostained with the conventional staining method described in “Conventional immunostaining method”. We used rat serum that is thought to contain about 10 mg/mL preimmune rat IgG as an isotype control (1:5000 dilution by filtered primary antibody diluent buffer), instead of using primary antibodies. No stained fibers were detected in rat serum treatment compared with the slides that applied with only primary antibody diluent or anti-MyHC2B antibody as a positive control. The bars indicate 200 μm.
(PPTX)

S1 Table Assessment of staining intensity against MyHCs after various fixation treatments. (DOCX)
