# Supplementary material for: A One-Step Immunostaining Method to Visualize Rodent Muscle Fiber Type within a Single Specimen
Source: PLoS One. 2016 Nov 4;11(11):e0166080. doi: 10.1371/journal.pone.0166080 (PMC5096669; doi:10.1371/journal.pone.0166080)
Supplement: S1 Fig — Slides containing muscle sections were immunostained with the conventional staining method described in “Conventional immunostaining method”. We used rat serum that is thought to contain about 10 mg/mL preimmune rat IgG as an isotype control (1:5000 dilution by filtered primary antibody diluent buffer), instead of using primary antibodies. No stained fibers were detected in rat serum treatment compared with the slides that applied with only primary antibody diluent or anti-MyHC2B antibody as a positive control. The bars indicate 200 μm. (PPTX) [file pone.0166080.s001.pptx]

## Slide 1
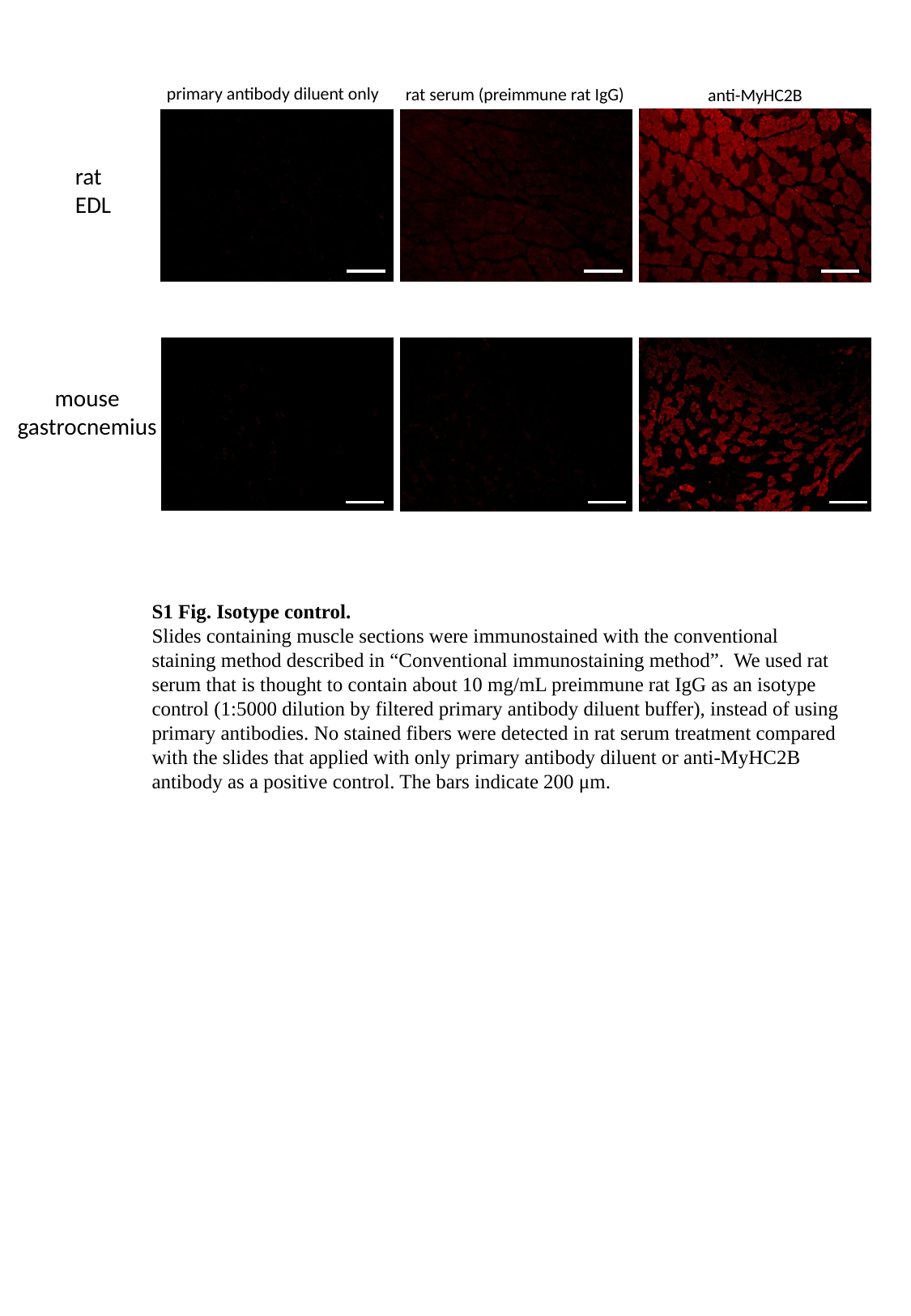

primary antibody diluent only
rat serum (preimmune rat IgG)
anti-MyHC2B
rat
EDL
mouse
gastrocnemius
S1 Fig. Isotype control.
Slides containing muscle sections were immunostained with the conventional staining method described in “Conventional immunostaining method”. We used rat serum that is thought to contain about 10 mg/mL preimmune rat IgG as an isotype control (1:5000 dilution by filtered primary antibody diluent buffer), instead of using primary antibodies. No stained fibers were detected in rat serum treatment compared with the slides that applied with only primary antibody diluent or anti-MyHC2B antibody as a positive control. The bars indicate 200 μm.
