# Supplementary material for: A One-Step Immunostaining Method to Visualize Rodent Muscle Fiber Type within a Single Specimen
Source: PLoS One. 2016 Nov 4;11(11):e0166080. doi: 10.1371/journal.pone.0166080 (PMC5096669; doi:10.1371/journal.pone.0166080)
Supplement: S1 Table — (DOCX) [file pone.0166080.s002.docx]

| S1 Table |  | |  | |
| --- | --- | --- | --- | --- |
| **Assessment of staining intensity against MyHCs after various fixation treatments.** | | | | |
|  | | 1% Triton X-100 | | |
|  |  | treated | | non-treated |
| 1. steam treatment in PBS | | +++ | | +++ |
| 2. acetone treatment | | - | | - |
| 3. PFA treatment | | - | | - |
| 4. PFA treatment  +steam treatment in citrate buffer | | + | | + |
| 5. boiling in PBS solution | | +++ | | +++ |
| 6. boiling in citrate buffer | | + | | + |
| 7. no fixation | | - | | - |
| * Staining intensity was scored as - (negative), + (weak), or +++ (strong). | | | | |

**1. steam (PBS):** steam for 5 min in PBS by food steamer, cool down for at least 30 min

**2. acetone:** immerse in aceton for 60 sec at -20°C and air-dry for 10 min at room temperature

**3. PFA:** immerse in 4% paraformaldehyde (PFA) in PBS for 20 min at 4°C

**4. PFA + steam (citrate buffer):** immerse in 4% PFA for 20 min at 4°C, then steam for 20 min in 10 mM citrate buffer (pH 6), then cool down for at least 30 min.

**5. microwave (PBS):** heat for 5 min in 200 mL PBS by microwave, leave for 5 min, heat for 5 min again, cool down for at least 30 min.

**6. microwave (citrate buffer):** heat for 5 min in 200 mL 10 mM citrate buffer (pH 6) by microwave, leave for 5 min, heat for 5 min again, cool down for at least 30 min.
